# Scaffold-free generation of uniform adipose spheroids for metabolism research and drug discovery

**DOI:** 10.1038/s41598-017-19024-z

**Published:** 2018-01-11

**Authors:** Aloysius J. Klingelhutz, Francoise A. Gourronc, Anna Chaly, David A. Wadkins, Anthony J. Burand, Kathleen R. Markan, Sharon O. Idiga, Meng Wu, Matthew J. Potthoff, James A. Ankrum

**Affiliations:** 1 University of Iowa Fraternal Order of Eagles Diabetes Research Center, 169 Newton Rd, Iowa City, IA 52242 USA; 2 Department of Microbiology, Carver College of Medicine, University of Iowa, Iowa City, IA 52242 USA; 3 Department of Biomedical Engineering, University of Iowa, Iowa City, IA 52242 USA; 4 Department of Pharmacology, Carver College of Medicine, University of Iowa, Iowa City, IA 52242 USA; 5 Department of Biochemistry, Carver College of Medicine, University of Iowa, Iowa City, IA 52242 USA; 6 Division of Medicinal and Natural Products Chemistry, Department of Pharmaceutical Sciences and Experimental Therapeutics, College of Pharmacy, University of Iowa, 115 S. Grand Ave, Iowa City, IA 52242 USA; 7 High Throughput Screening Core Facility at University of Iowa (UIHTS), University of Iowa, 115 S. Grand Ave, Iowa City, IA 52242 USA

## Abstract

Adipose tissue dysfunction is critical to the development of type II diabetes and other metabolic diseases. While monolayer cell culture has been useful for studying fat biology, 2D culture often does not reflect the complexity of fat tissue. Animal models are also problematic in that they are expensive, time consuming, and may not completely recapitulate human biology because of species variation. To address these problems, we have developed a scaffold-free method to generate 3D adipose spheroids from primary or immortal human or mouse pre-adipocytes. Pre-adipocytes self-organize into spheroids in hanging drops and upon transfer to low attachment plates, can be maintained in long-term cultures. Upon exposure to differentiation cues, the cells mature into adipocytes, accumulating large lipid droplets that expand with time. The 3D spheroids express and secrete higher levels of adiponectin compared to 2D culture and respond to stress, either culture-related or toxin-associated, by secreting pro-inflammatory adipokines. In addition, 3D spheroids derived from brown adipose tissue (BAT) retain expression of BAT markers better than 2D cultures derived from the same tissue. Thus, this model can be used to study both the maturation of pre-adipocytes or the function of mature adipocytes in a 3D culture environment.

## Introduction

Adipose function and signalling play critical roles in the development of type II diabetes and other metabolic diseases. There is a significant need for a thorough understanding of how fat tissue becomes dysfunctional and for the development of therapeutics to prevent and reverse this process. A major limiter to meeting this need is a lack of models that faithfully reproduce human physiology and pathophysiology. Monolayer cell culture systems have been useful for understanding many aspects of fat biology, but these 2D *in vitro* systems do not reflect the complexity of human fat tissue. While animal model systems more closely resemble human conditions, they are expensive, time consuming, and due to species variation do not always faithfully predict human biology^1–3^. To address these problems, we have developed a facile method to fabricate hundreds of 3D adipose spheroids from human and mouse pre-adipocytes that exhibit morphological and physiological behaviours that mimic *in vivo* adipose tissue.

Adipose tissue has emerged as a key player in the development of type II diabetes, cardiovascular disease, and cancer^4–7^. Adipocytes do much more than store triglycerides, and “healthy” adipose tissue is critical for maintaining normal metabolism. Healthy mature white adipocytes secrete numerous cytokines and hormones (adipokines) such as adiponectin and leptin that have endocrine effects on tissues throughout the body^8^. Dysfunctional adipocytes secrete reduced levels of adiponectin and leptin and can become pro-inflammatory^9,10^. Excessive release of free fatty acids (FFAs) by dysfunctional adipocytes is associated with the development of insulin resistance^11^ and can cause fat accumulation in other tissues (i.e. liver steatosis)^12^.

White adipocyte dysfunction is multifactorial. The classic model is that accumulation of triglycerides in adipocytes causes them to stretch to their limit and become hypertrophic, inflammatory, and nonfunctional^7^. Hypertrophic obesity is also associated with an inability to recruit and differentiate new adipocytes as old adipocytes die^13^. As adipose tissue becomes hypertrophic and crowded, a shift to an adverse adipokine profile occurs which includes an elevated level of pro-inflammatory cytokines with a parallel reduction in anti-inflammatory factors such as adiponectin^14^.

Because of normal cell death processes (apoptosis, hypertrophy, etc.), approximately 10% of adipocytes in the human body are replaced every year^15^. This replacement relies on adipocyte precursor cells called pre-adipocytes, which originate from mesenchymal stem cells (MSCs). Because adipocytes need to be replaced, disruption of adipogenesis can also lead to dysfunctional adipose tissue. Certain drugs such as the thiazolinediones (the glitazones) increase adipogenesis through activation of the adipocyte-specific transcription factor PPARγ and have been used for treatment of type II diabetes because they are insulin sensitizers^7^. Recent studies indicate that transplanting healthy adipocytes into diabetic mice can significantly improve obesity-associated hyperglycemia^16,17^. In addition to diet induced obesity and diabetes, certain chemicals in the environment and bacterial toxins are also known to affect both adipogenesis and adipocyte function, potentially leading to obesity and/or diabetes^18,19^. For example, we have found that the dioxin-like polychlorinated biphenyl (PCB) 126 inhibits adipogenesis with concomitant downregulation of PPARγ, and this is associated with a strong pro-inflammatory phenotype^20^.

Because of the clear roles for adipose tissue in health and disease, there has been a growing interest in identifying and developing therapeutic drugs that target adipose function. Most *in vitro* studies on adipocytes have been performed using mouse 3T3-L1 cells^21^. However, due to differences in the way that mice and humans metabolize drugs and their sensitivity and response to different agents^22^, it is important that human cells be utilized in preclinical testing. Furthermore, because cells in 2D culture are under vastly different microenvironmental and physical conditions than *in vivo* tissue, cell morphology and function of 2D adipocyte cultures do not mirror their *in vivo* counterparts.

Thus, there is a need to continue to improve models of adipose tissue in culture that more faithfully recapitulate fat’s *in vivo* phenotype that can be readily scaled to generate hundreds to thousands of microtissues using cells from a variety of mouse and human sources. Such a strategy would provide an *in vitro* tool for the discovery of drugs that modulate adipose function, or integration of adipose into multi-organ on a chip systems allowing for more accurate pharmacokinetic studies, as well as fundamental biological studies of adipose biology in the setting of metabolic diseases.

We theorized pre-adipocytes could be isolated and aggregated into scaffold-free spheroids using a hanging-drop technique previously described for other cells types^23,24^. We hypothesized the 3D environment would cause the cells to mature into adipocytes that more closely mirror *in vivo* adipocyte morphology and function as critical cell-cell cues that are absent in 2D cultures would be maintained. We selected a scaffold-free approach to determine if pre-adipocytes would generate sufficient matrix to support their own attachment and aggregation before increasing the complexity of the system through introduction of a foreign biomaterial. Finally, to demonstrate the utility of the adipose protocol, adipose spheroids were generated from both mouse and human sources and characterized for their native function as adipocytes as well as their response to stress cues and environmental toxins.

## Results

### Hanging Drop Method Generates Uniform Adipose Spheroids That Expand in Culture

**Figure 1:**
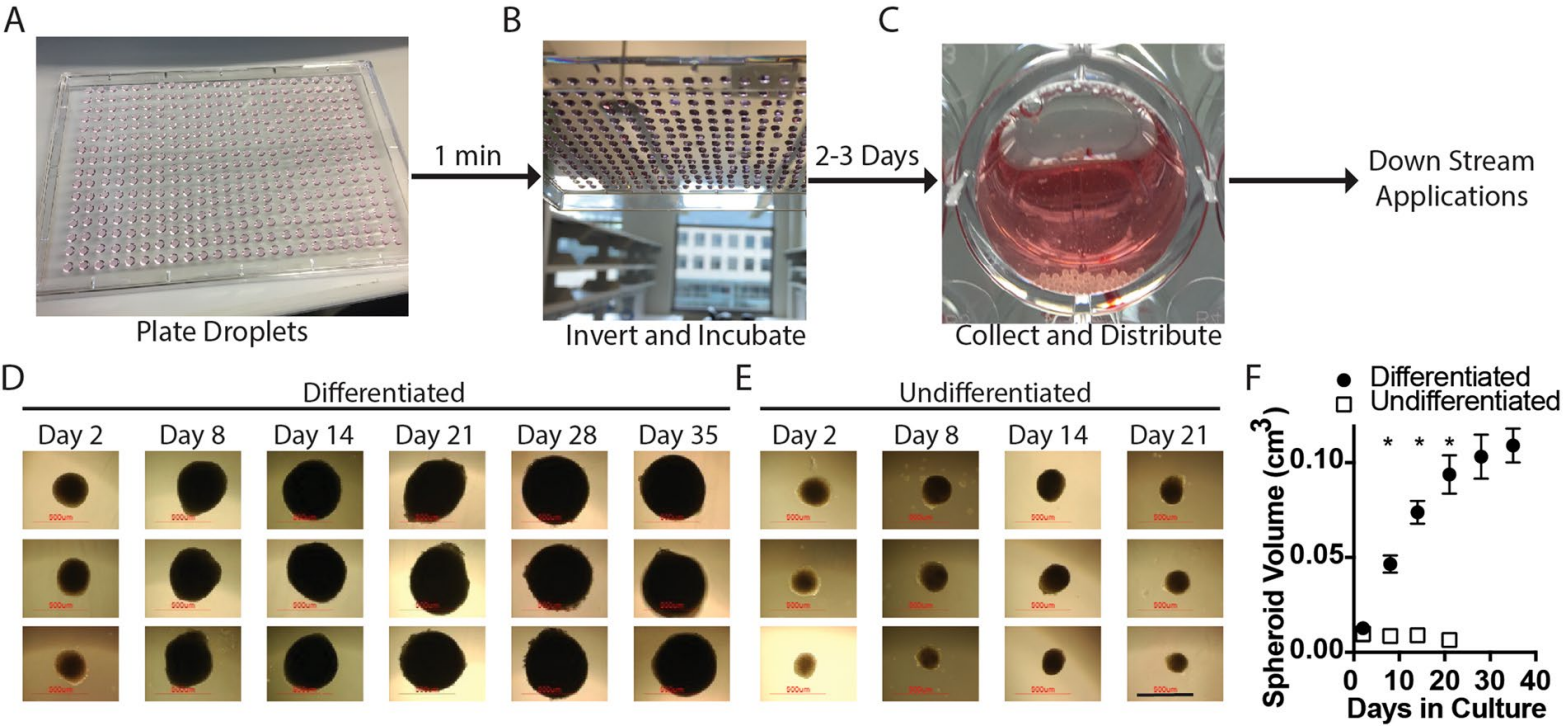
Scaffold-free generation of adipose spheroids that grow in culture when placed in differentiation media. (**A**) Suspensions of immortal human pre-adipocytes were deposited on the underside of the lid of a 384 well plate using an Integra Viaflo Assist robotic pipette in 20 µl droplets. (**B**) The lid was then inverted to create hanging drops and the plate was incubated at 37 °C in a humidified chamber for 2–3 days to allow for self-assembly of pre-adipocytes into spheroids. (**C**) The resultant spheroids were then gently washed from the lid of the plate and collected into the wells of 24 well ultra-low binding plates for downstream applications. In some cases, single spheroids were placed in 96 well plates. (**D**) Phase-contrast image of spheroids plated singly into wells over 35 days in adipocyte differentiation media reveal growth of the spheroids. (**E**) Spheroids maintained in base media showed no appreciable change in size after 3 weeks of observation. (scale = 500 µm) (**F**) Quantification of the volume of spheroids cultured in base media versus adipocyte differentiation media (Bars are mean +/− SD, 2-Way ANOVA with media condition and days in culture as independent variables, n = 3 for undifferentiated samples and n = 6 biological replicates for differentiated samples, *denotes significant difference (p < 0.05) between media condition at a given time point after Sidak correction for multiple comparisons). After 21 days in culture, undifferentiated spheroids lost their integrity and size measurements were halted.

We wanted to develop a 3D system that would allow cell expansion that would more closely resemble *in vivo* conditions. To do this, we took advantage of the ability of pre-adipocytes to form spheroids when forced together in hanging drops. To set up the system, we initially used immortalized human subcutaneous pre-adipocytes that we have previously described^25–27^. The cells are referred to as NPADs (normal pre-adipocytes) and were derived from a non-diabetic donor. They are a stable cell line that has the ability to differentiate *in vitro*, exhibiting proper markers of adipocyte differentiation^25–27^. NPAD pre-adipocyte droplets consisting of 1 million cells per milliliter of pre-adipocyte growth media were deposited on the underside of the lid of a cell culture plate using a multichannel pipette at 20 µl (20,000 cells) per droplet (Fig. 1A). The lid was then inverted and placed on a tissue culture plate containing PBS (for humidity) for two days to allow spheroid formation (Fig. 1B). After 2 days, the spheroids were transferred to ultra-low adherent wells (to prevent attachment) in differentiation or non-differentiation media (Fig. 1C). When in differentiation media, the spheroids expanded over time (Fig. 1D). Quantitation of spheroid volume demonstrated that those spheroids in differentiation media expanded whereas those in non-differentiating growth media did not (Fig. 1E). These results indicate that the spheroid volume increases over time, much like would be expected for adipose tissue that accumulates lipid as pre-adipocytes mature into adipocytes.

### Adipose Spheroids Develop Unilocular Lipid Droplets as they Mature

**Figure 2:**
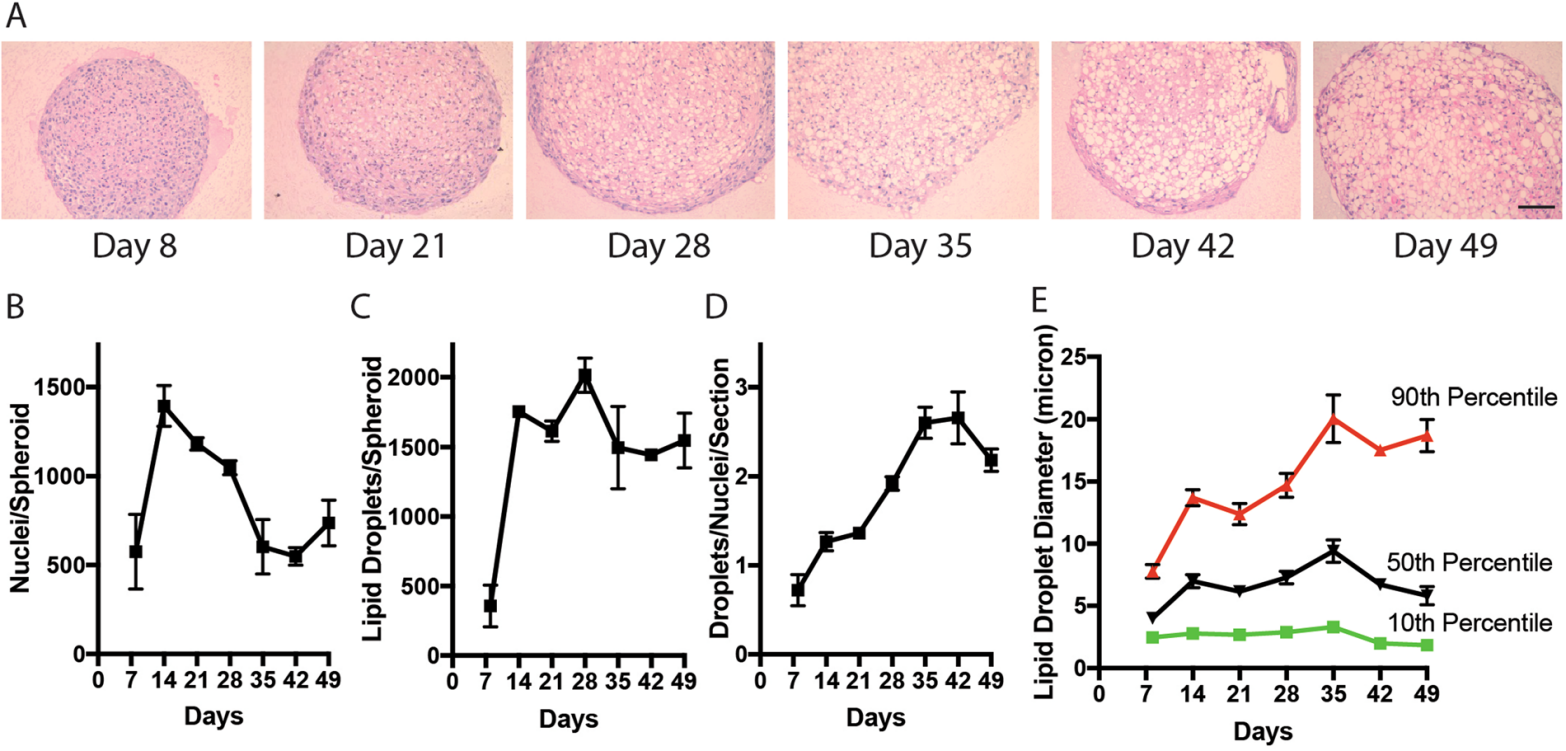
Spheroid growth in differentiation media is caused by accumulation and growth of lipid droplets. (**A**) H&E stained histology images taken from 10-micron slice of spheroids (derived from immortal human pre-adipocytes) harvested after 8–49 days of maintenance in differentiation media with media changed every week. Spheroids were maintained as singlets in 96-well ultra-low binding plates (scale = 100 µm). Quantification of (**B**) nuclei per section (**C**) number of lipid droplets per section (**D**) Droplet/nuclei ratio per section, and (**E**) diameter of lipid droplets in each section of representative adipose spheroids over time in culture.(mean +/− SEM, 4–6 Spheroids analysed at each time point).

Next, to determine if the apparent growth of the differentiated spheroids was due to cell proliferation or accumulation of lipid droplets, a series of imaging studies were performed. Lipid drop accumulation was assessed by fixing the spheroids, embedding in paraffin, sectioning and H&E staining (Fig. 2A). Expansion is accompanied by a decrease in cell number (Fig. 2B) and an accumulation of lipid droplets (Fig. 2C) that increase in size (Fig. 2E) with time throughout the spheroid. After 30 days in differentiation media, the spheroids stained brightly for both AdipoRed and perilipin confirming the droplet structures observed on H&E were indeed lipid droplets (Fig. S1). Thus, the spheroid system provides an *in vitro* model of pre-adipocyte maturation and adipocyte expansion. In 2D culture systems of adipogenesis, lipid drop formation occurs but is multilocular in nature whereas mature white adipocytes *in vivo* exhibit unilocular lipid drops that expand with time^7^. While the ratio of lipid droplets to nuclei across the entire spheroid never reaches 1, large regions of the spheroid cross section develop what appear to be large unilocular lipid droplets. The upper 10^th^ percentile of fat droplets in spheroids cultured in differentiation media have diameters in excess of 20 µm. Thus, the presence of a large number of unilocular droplets in our adipose spheroid system provides morphological evidence of adipogenic differentiation.

### Hanging Drop Technique is Adaptable to Different Cell Sources

**Figure 3:**
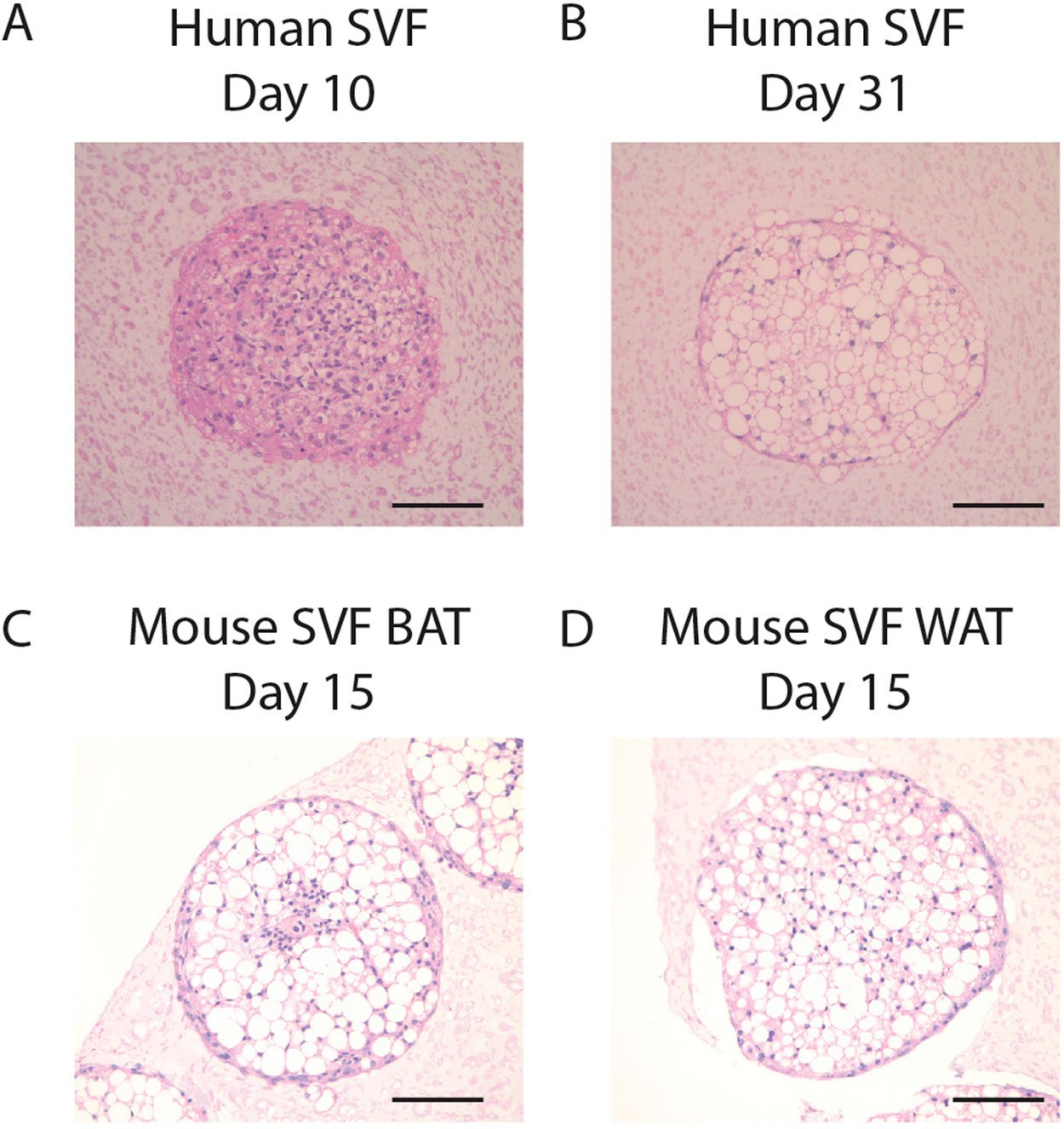
Scaffold-free adipose spheroid method is compatible with primary human and mouse primary stromal vascular fractions. (**A**) Human stromal vascular fraction (SVF) coalesces into spheroids in hanging drop cultures. (**B**) Human SVF spheroids accumulate large unilocular lipid droplets over time in differentiation media in culture. (**C**) Representative image of spheroid generated from mouse SVF from brown adipose tissue after 15 days of culture in differentiation media. (**D**) Representative image of spheroid generated from SVF of mouse white adipose tissue after 15 days of culture in differentiation media. (scale = 100 µm).

While the hanging drop spheroid system with immortalized pre-adipocytes showed vast improvements in morphology compared to previously reported adipose culture systems, we wanted to determine whether the spheroid system was amendable to primary cells harvested from stromal vascular fraction (SVF) isolated from adipose tissue. These cells are mixed cultures and have a notoriously short lifespan of only a few passages but can be propagated to high enough numbers to generate hanging drops. We used SVF from subcutaneous fat derived from discarded skin tissue within 2 passages after isolation. Spheroids derived from these cells again gave rise to differentiated adipocytes with many unilocular lipid droplets (Fig. 3A and B). Like the immortalized spheroids, SVF based spheroids cultured in differentiation media, but not PGM2 base media, also expanded with time (Fig. 3B). Thus, SVF is amendable to spheroid generation that allows for the development of well-differentiated adipocytes within an intact 3D matrix produced entirely by the resident cells. In addition to human SVF, we also generated spheroids from white adipose tissue (WAT) and brown adipose tissue (BAT) from mouse neonates. Cells in spheroids from these tissues also readily differentiated into mature adipocytes with large lipid droplets (Fig. 3C and D). Interestingly, the morphology of spheroids derived from mouse BAT SVF consistently displayed a region of elevated cell density lacking lipid accumulation in the core of the spheroid. Collectively, these results demonstrate the robust utility of the spheroid culture system to generate 3D mature adipocyte tissues from both primary and immortalized, human and mouse cell sources. Such a system could provide a much-needed *in vitro* assay to study adipogenesis, adipocyte expansion, and adipose tissue pathology as we demonstrated next.

### Adipose Spheroids are Environmentally Sensitive

**Figure 4:**
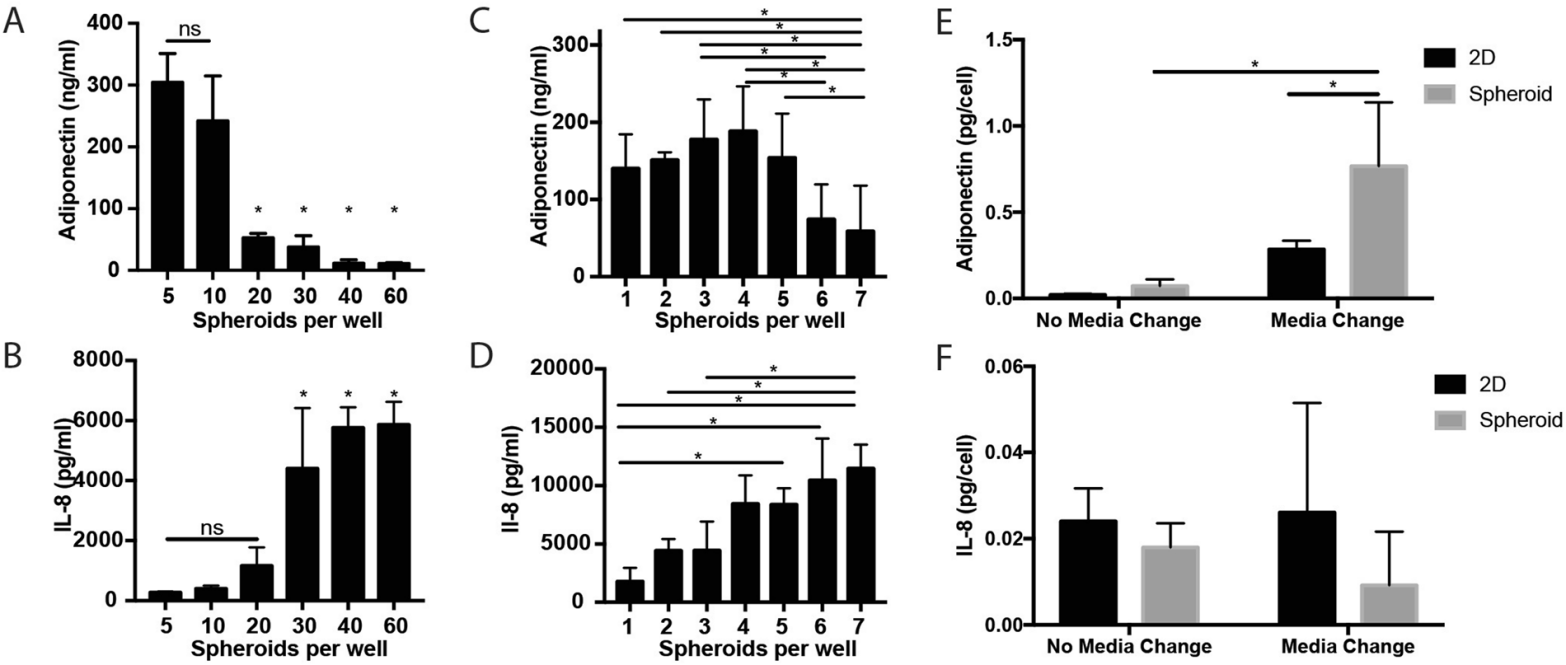
Adipose spheroids are sensitive to overcrowding and produce significantly higher amounts of adiponectin than their 2D counterparts. (**A**) Adiponectin levels drop while (**B**) IL-8 levels spike when the number of spheroids cultured together in a well increases from 5 to 60. (**C**) A follow up experiment showed the sensitivity exists at lower seeding densities as well with adiponectin secretion peaking at 4 spheroids per well while (**D**) IL-8 secretion increases with each addition of a spheroid. (**E**) Comparison of adiponectin and (**F**) IL-8 production over a 24-hour period after 10 days in culture with media changes every 3^rd^ day using cells seeding in 2D tissue culture plastic or as adipocyte spheroids in ultra-low binding plates. All experiments were done in 24-well plates using immortal human pre-adipocytes. (**A**–**D**), 1-Way ANOVA, (**A**) *denotes significant difference (n = 3, p < 0.05) between the marked group and both groups 5 and 10 after Tukey correction for multiple comparisons (**B**) *denotes significant difference (n = 3, p < 0.05) between the marked group and groups 5, 10, and 20 after Tukey correction for multiple comparisons. (**C**,**D**) *Denotes significant difference (n = 6, p < 0.05) after Tukey correction for multiple comparisons. (**E**,**F**) (2-Way ANOVA with media condition and 2D/Spheroid as independent variables, *denotes significant difference (n = 6, p < 0.05) Tukey correction for multiple comparisons).

To be able to use the adipose spheroid system as a model to study adipose dysfunction, we wanted to determine if the spheroids were biologically active in secreting adipose specific factors such as adiponectin, a hormone that is secreted by mature adipocytes. To determine the optimal number of spheroids/well for biological assays, we placed 5–60 spheroids in each well of a 24-well plate and examined their production of adiponectin in the media after 10 days differentiation. We found an inverse relationship between the number of spheroids per well and the level of adiponectin secretion (Fig. 4A). This is likely due to the generation of metabolic stress in wells with higher numbers of spheroids as the higher cell density could both deplete nutrients while at the same time releasing anabolites into the media. A similar phenomenon of adipocyte crowding leading to adipose dysfunction is seen in the setting of obesity^14^. Knowing adipocytes *in vivo* respond to stress by suppression of adiponectin secretion and increases in cytokine production, we assayed the levels of the pro-inflammatory cytokine IL-8 in the conditioned media. Interestingly, levels of IL-8 were opposite of those of adiponectin, increasing with spheroid number rather than decreasing (Fig. 4B). These results indicate that having too many spheroids in a well causes stressful conditions that reduce adiponectin secretion. In further experiments, we found that 3–5 spheroids/well with media changes every 3 days gave the highest levels of detectable secreted adiponectin (Fig. 4C and D) and used 3 spheroids per well for all subsequent experiments.

To further test the hypothesis that metabolic stress led to less adiponectin secretion, we examined the effect of the frequency of media changes on adiponectin secretion. On a per cell basis, the cultures in which media was changed every 3 days secreted more adiponectin than those that were media changed less frequently (Fig. 4E). These findings are of particular interest since it has been well-documented that adipose tissue becomes pro-inflammatory when intracellular stress and inflammation pathways are activated, including oxidative stress and ER stress pathways and lower levels of adiponectin secretion are associated with this pro-inflammatory state^7,28,29^. Thus, the spheroid system may be useful for studying the relationship between stress, nutrient availability, inflammation, and abrogation of biological function in adipocytes.

### Adipose Spheroids Have Enhanced Adiponectin Secretion Compared to 2D Culture

We were interested in determining how the biological function of the 3D adipose spheroids compared to 2D culture. To test this, we established cultures in which the same number of cells from hanging drops (3 hanging drops) were transferred into the wells of either regular culture plates (2D) or ultra-low adherent plates (3D). The cells were differentiated for 10 days. At the end of the differentiation period, we measured levels of adiponectin. On a per cell basis (as quantified by nucleic acid content), the 3D spheroids secreted over 4X as much adiponectin into the media compared to 2D cultures with little difference in IL-8 secretion (Fig. 4E and F). This result indicates that the spheroids are biologically active and adipocytes in 3D culture produce significantly more adiponectin compared to their 2D counterparts.

### Spheroids from white and brown adipose maintain differential gene expression

**Figure 5:**
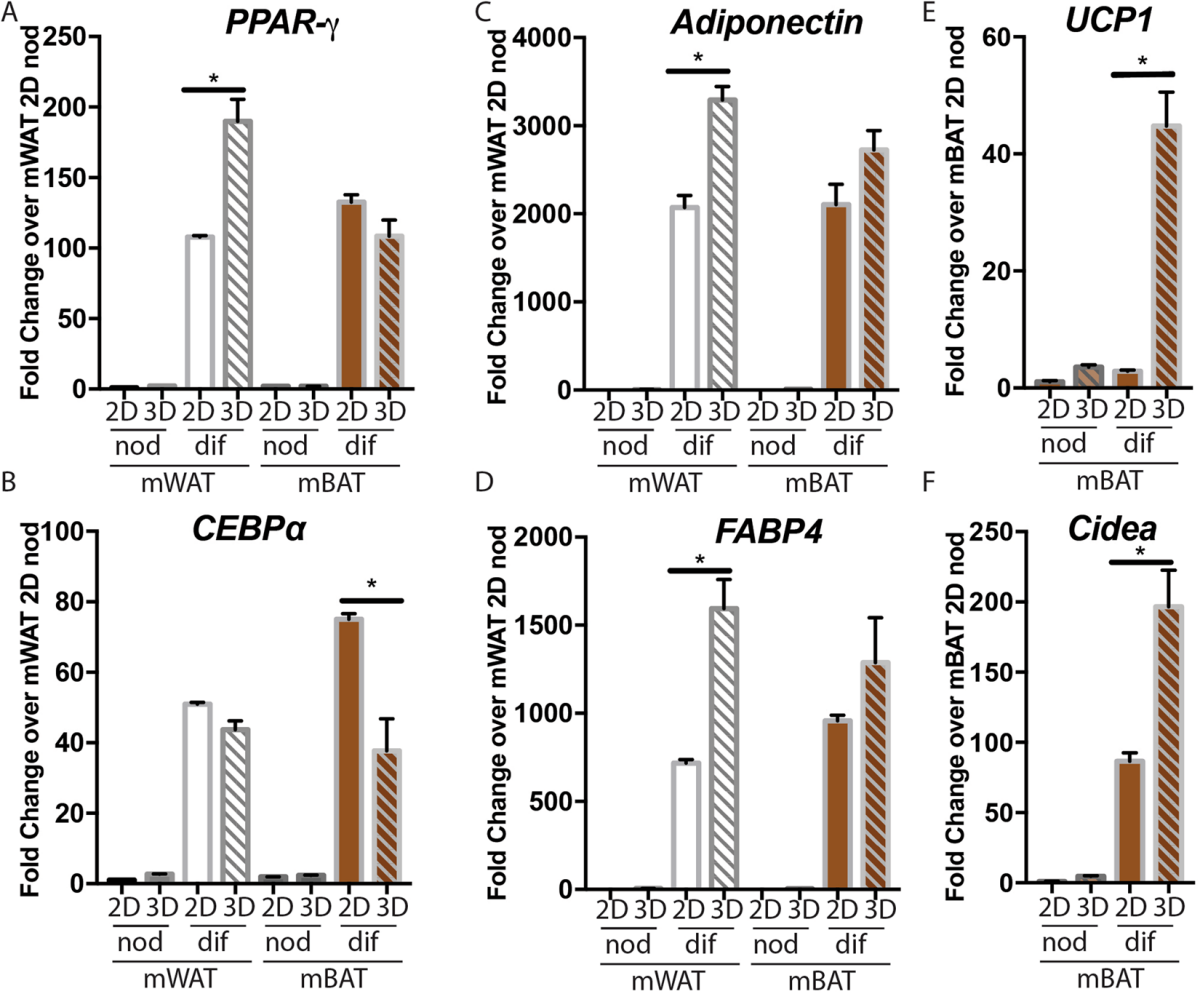
Adipose spheroids maintain white and brown adipose characteristics. Quantitative RT-PCR (qRT-PCR) of (**A**) *PPAR-γ* (**B**) *CEBPα* (**C**) *Adiponectin* (**D**) *FABP4* transcripts in mWAT and mBAT cultured in 2D tissue culture plastic or 3D, spheroid cultures in non-differentiation (nod) or differentiation media (dif) (**E**) *UCP1 and* (**F**) *Cidea* transcripts in mouse BAT cultured in non-differentiating (nod) or differentiating (dif) media in 2D or 3D (spheroid) conditions. WAT or BAT tissue was isolated from mouse pups and the stromal vascular fraction cultured as described in the Materials and Methods. Values represent fold change over non-differentiated WAT in 2D culture for (**A**–**D)** and non-differentiated BAT in 2D culture for (**E**) and (**F**). (Bars are mean +/− SEM, Ordinary One-way ANOVA used to compare 2D versus 3D adipocytes from the same tissue source and media condition with Sidak correction for multiple comparisons, n = 3, *p < 0.05).

We wanted to determine whether spheroids derived from WAT or BAT retained their differential gene expression and how this compared to 2D culture. Spheroids and 2D cultures derived from mouse primary WAT and BAT SVF were either differentiated or held in non-differentiating conditions after which RNA was isolated and assessed by qRT-PCR for transcript levels of markers of adipocyte differentiation, including the adipogenesis transcription factors *PPAR*γ and *CEBPα*, and the adipocyte differentiation markers *adiponectin* and *FABP4* (fatty acid binding protein 4), as well as the brown adipocyte markers *UCP1* and *Cidea*^30^. As long term cultures of adipocytes in 2D are difficult to maintain, RNA was harvested from both spheroid and 2D cultures after 7 days in culture. WAT spheroids expressed significantly higher levels of *PPAR*γ, the master transcriptional regulator of adipogenesis (Fig. 5A). Similar levels of the transcription factor, *CEBPα*, were measured in the 2D and Spheroid WAT cultures and surprisingly showed a significant increase in 2D BAT vs. BAT spheroids exposed to differentiation media (Fig. 5B). Looking at later markers of adipocyte differentiation, we found WAT spheroids expressed significantly higher levels of both *adiponectin* and *FABP4* transcripts than differentiated 2D cultures while BAT spheroids expressed similar levels in both 2D and spheroid cultures (Fig. 5C and D)^31,32^. It should be noted that while significant differences between 2D and spheroid were seen for *PPAR*γ, *CEBPα*, *adiponectin*, and *FABP4*, all increased in response to exposure to differentiation media and differences between 2D and spheroid was less than 2-fold in each case.Looking specifically at brown adipocyte markers *UCP1* and *Cidea*, we found significantly greater expression in spheroids compared to 2D cultures (Fig. 5E and F) with an over 20-fold increase in *UCP1* transcript in spheroids compared to 2D BAT cultures. Thus, the spheroids derived from WAT and BAT continue to express differentiation markers of their tissue source at levels comparable or higher than cells from the same tissues grown in 2D cultures, a potentially useful property for studies of factors that regulate browning.

### Spheroids are sensitive to environmental toxins, upregulating cytokine production and down regulating adiponectin secretion in response

**Figure 6:**
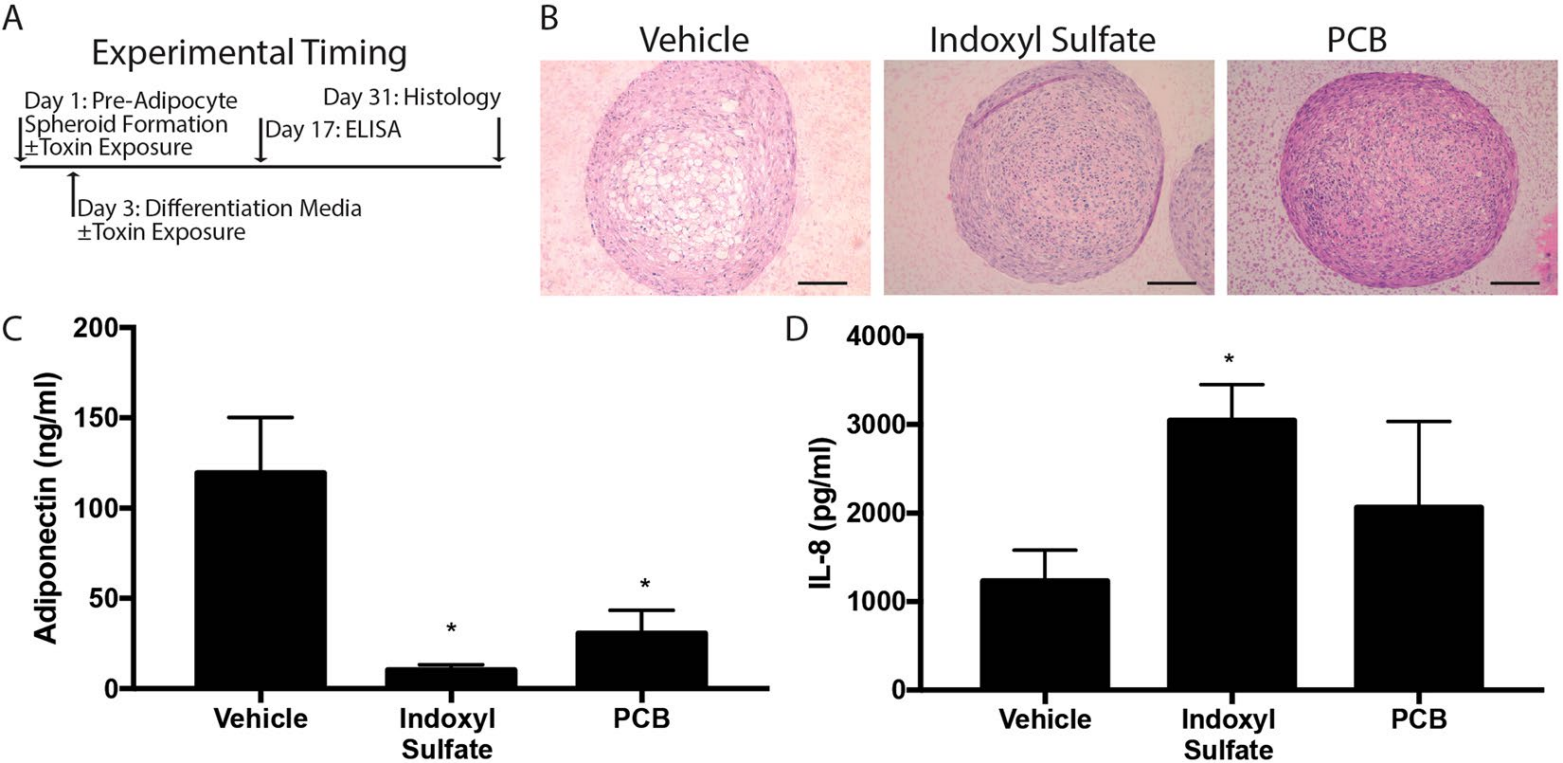
Adipose spheroids are sensitive to environmental toxin exposure during differentiation. (**A**) Experimental timing of spheroid formation, toxin exposure, differentiation, and analysis. (**B**) H&E stained histology images taken from 10-micron slice of spheroids harvested after 31 days of maintenance in differentiation media with or without indoxyl sulfate or PCB, both at 10 μM, with media changed every 3^rd^ day. (scale = 100 µm). (**C**) Adiponectin and (**D**) IL-8 measured in media collected on day 17 of differentiation/toxin exposure. Spheroids were made using immortal human pre-adipocytes with 3 spheroids/well in 24-well plates. 1-Way ANOVA, n = 6 biological replicates, *denotes significant difference (p < 0.05) between the marked group and vehicle (DMSO) treated spheroids after Sidak correction for multiple comparisons.

Next, we sought to prove the concept that the adipose spheroid system could be used as a tool to model the response of adipose tissue to environmental toxins. Environmental and bacterial toxins have been shown to affect adipogenesis and adipose inflammation^33,34^. Our previous studies demonstrated that aryl hydrocarbon receptor (AhR) agonists such as certain polychlorinated biphenyls (PCBs) are capable of inhibiting adipogenesis^20,35^. Other studies have shown that the microbiome-derived metabolite, indoxyl sulfate, a tryptophan derivative, acts as an AhR agonist^36^. We were interested in determining how these compounds affected spheroid function as a proof-of-concept that spheroids can be used as a drug screening assay. Hanging drops were generated from pre-adipocytes and transferred to ultra-low adherent plates in base PGM2 media containing vehicle, PCB126, or indoxyl sulfate for 2 days followed by addition of differentiation media. After 2 weeks of differentiation, supernatants were collected and after 4 weeks differentiated spheroids were fixed for H&E staining (Fig. 6A). Spheroids that were differentiated in PCB126 or indoxyl sulfate showed both morphological and phenotypic changes. Morphologically, toxin treated spheroids had fewer and smaller lipid droplets, suggesting that adipogenesis was impaired (Fig. 6B). In addition, levels of secreted adiponectin were significantly down while levels of IL-8 were up in spheroid cultures treated with either PCB126 or indoxyl sulfate (Fig. 6C and D). Thus, the AhR agonists caused a pro-inflammatory response and concomitantly inhibited adiponectin secretion from the spheroids. While many other environmental and metabolic factors are known to affect adipocyte function, this data shows adipose spheroids are sensitive to changes in their environment and amenable to culture studies lasting days to weeks. This is a feat that is challenging to achieve with traditional 2D cultures due to spontaneous lifting of differentiated cells after long-term culture. Thus, the spheroids provide a system to assess the long-term effects of different factors on adipocyte function.

### Adipose spheroids are suitable for high throughput screening platforms

**Figure 7:**
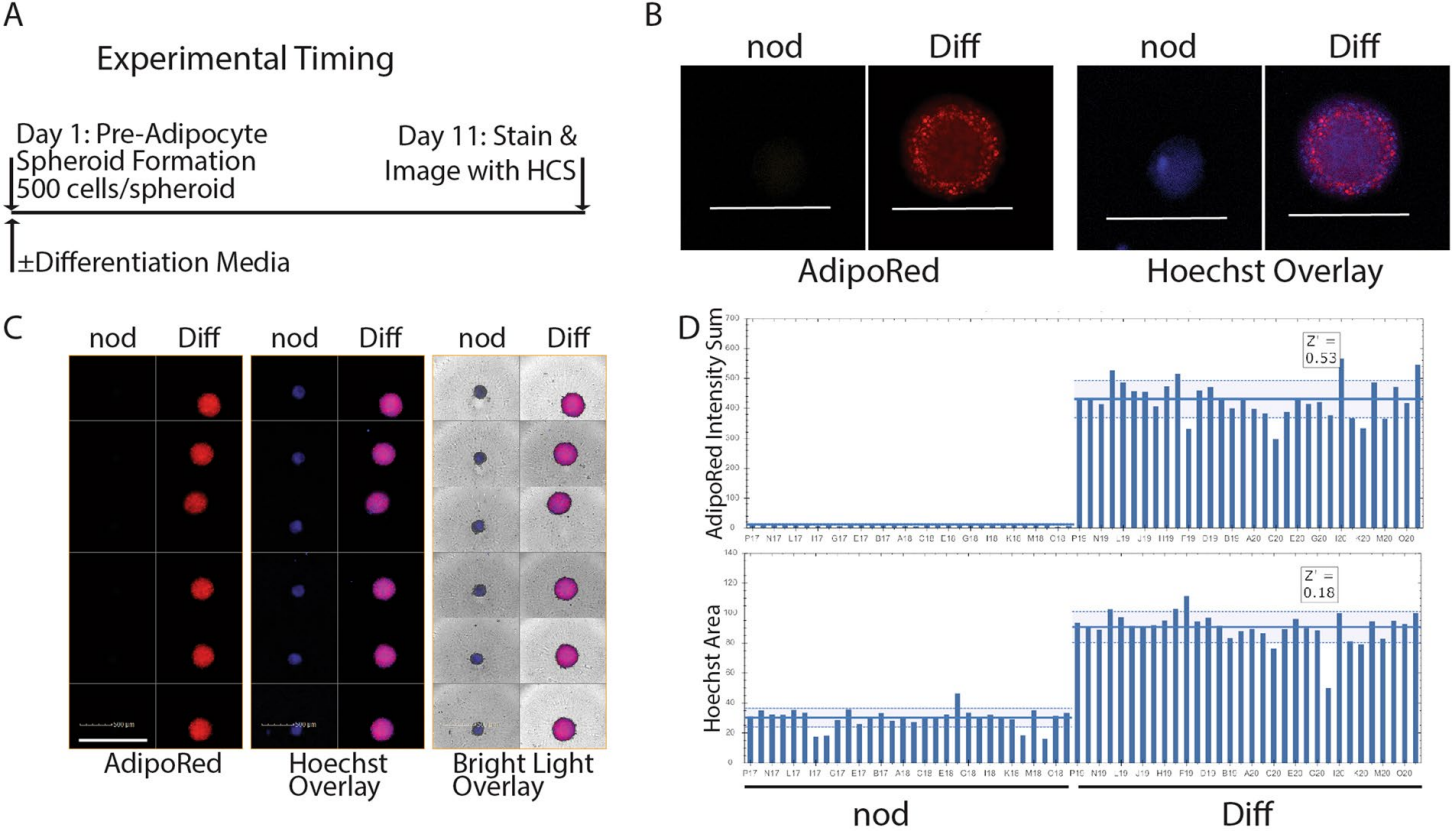
Adipose spheroids can be adapted to high throughput screening platforms. (**A**) Experimental timing of spheroid formation, differentiation, and analysis in ultra-low attachment spheroid plates. (**B**) Confocal slice through the center of an un-differentiated (nod) and differentiated (Diff) adipose spheroid stained with Hoechst (Blue) and AdipoRed (Red), scale bar = 500 µm (10× LWD, Operetta HCI system, one of 12 stacks at 110 µm). (**C**) Epifluorescence images of 12 undifferentiated and 12 differentiated adipose spheroids stained with Hoechst (Blue) and AdipoRed (Red), scale bar = 1000 µm (10× LWD, Operetta HCI system). (**D**) Quantification of AdipoRed intensity sum and Hoechst Area of n = 30 undifferentiated and n = 30 differentiated spheroids in different wells using Harmony. All spheroids were made using immortal human pre-adipocytes.

To determine if the adipose spheroid platform is compatible with established drug screening tools, we adapted our protocol to 384-well plate format traditionally used for high throughput screening (Fig. 7A). Using a plate treated for ultra-low attachment and a round bottom, highly uniform spheroids could be readily formed in the plates. Like hanging drops, the conical shape of the plates caused cells to aggregate into spheroids and spheroids could be directly differentiated in the plates. High content image analysis of the plates using both confocal (Fig. 7B) and epifluorescence (Fig. 7C) could then be used to quantify the size and lipid content of the spheroids after staining with nuclear (Hoechst) and lipophilic (AdipoRed) dyes respectively (Fig. 7D). Induction of differentiation in pre-adipocytes is known to result in temporary re-entry into the cell cycle and a doubling in cell number before differentiation begins^37^. This is validated by our finding that the differentiated spheroids have 2-fold more Hoechst signal than non-differentiated spheroids (Fig. 7D). AdipoRed signal, on the other hand, is approximately 200-fold more in the differentiated spheroids. This proof-of-concept study showed adipose spheroid differentiation efficiency could be monitored using high content imaging with AdipoRed intensity sum measurements resulting in the ability to efficiently distinguish between lipid rich, differentiated spheroids from their undifferentiated counterparts (Z’ = 0.53, n = 30 per condition).

## Discussion

Adipocytes have an extraordinary ability to increase in size through accumulation of lipids^27^. This ability is important for energy storage but, in obesity, can lead to hypertrophy and conversion to a pro-inflammatory state^29^. Two-dimensional culture is not an ideal situation for culture of adipocytes because of limitations on growth and the accumulation of small, multi-locular lipid droplets. Curiously, most previous efforts to generate 3D adipose cultures have relied on the use of exogenous scaffolding material to aggregate cells together^38–40^. While successful in creating aggregates, scaffold based strategies introduce additional complexity based on the assumption that the pre-adipocytes and SVF cells are not capable of producing their own supportive matrix. This assumption, however, is false, as it is known that pre-adipocytes grown in suspension cultures or non-adherent surfaces self-assemble into spheroids^41–44^. Adipocytes in these cultures accumulate more lipid than their 2D counterparts and show increased expression of adipogenic markers. However, these strategies fail to create microtissues that mirror both the functional (adiponectin secretion, environmental sensitivity) and histological characteristics (large, unilocular lipid droplet accumulation) of native adipose tissue. Notably most efforts to date have utilized either immature adipose derived stem cells^39,41,42^, pre-adipocytes^40^, or 3T3-L1 mouse pre-adipocytes^43^ possibly contributing to the lack of unilocular lipid droplet morphology typically seen in mature adipocytes *in vivo*. To date, the closest generation of a 3D adipose microtissue has come from entrapping mature adipocytes within a scaffold. Abbott *et al*. soaked silk scaffolds in liquefied adipose lipoaspirate resulting in the entrapment of mature adipocytes within the pores of the scaffold network^38^. The resultant microtissue maintained a unilocular morphology for 14 days in culture and maintained secretion of glycerol and accumulation of triglycerides to a degree similar to tissue explants^38^. While promising, reliance on exogenous materials and bulk manufacturing make producing large numbers of identical microtissues for high throughput screening challenging.

To overcome the challenges and shortcomings of previous attempts outlined above, we challenged the assumption that a scaffold was necessary to generate large numbers of uniform adipose spheroids. We found the scaffold-free approach presented here is robust, and able to generate hundreds of adipose spheroids that grow and mature in culture taking on the histological morphology and secretory phenotype of native adipocytes. We utilized a hanging drop spheroid method to generate uniform droplets of pre-adipocyte cell suspensions that self-organize in a controlled fashion into uniform sized spheroids. Once transferred to differentiation media, the spheroids grew in size as the pre-adipocytes matured into adipocytes and accumulated large lipid droplets throughout the thickness of the microtissue. Furthermore, the adipose spheroids displayed key similarities in both morphology and function to *in vivo* adipose tissue. The spheroids expressed and secreted adiponectin, a marker of mature adipocytes, at higher levels than differentiated 2D cultures. Additionally, spheroids developed from brown fat retained differential expression of *CIDEA* and *UCP1*, brown fat markers, better than cells grown in traditional 2D culture. Finally, the spheroids were responsive to environmental cues, exhibiting a reduction in adiponectin secretion associated with a pro-inflammatory response induced by culture stress or environmental toxins, as would be expected of adipose tissue.

While the current study was focused on developing and characterizing the adipose spheroid system, we envision this technique being tailored in the future for use in a number of applications. As the *in vitro* adipose spheroid system described here is compatible with primary human cells, it can readily be customized to model a specific patient demographic or individual without the need for immortalization. This is critical as both basic biology and drug discovery studies often suffer due to poor representation of females and ethnic minorities. The adipose spheroid system can be used to test hundreds to thousands of drug treatments using cells collected from a simple biopsy, allowing for rigorous and reproducible studies of the biology and drug response of adipose tissue derived from any patient group where a fat biopsy is accessible.

The techniques outlined here represent a facile method to generate uniform adipose microtissues that can be incorporated into studies of adipose biology (diabetes, obesity, metabolic disease), drug screening, or organ/person on a chip technologies. In contrast to previous attempts of building an *in vitro* adipose microtissue, our approach is scaffold-free, relying only on extracellular matrix proteins produced by the cells themselves. Additionally, adipocytes within our system consistently generated large numbers of unilocular droplets within spheroids derived from both immortalized and primary pre-adipocytes harvested from either mouse or human donors. The ability to mature the spheroids in culture enables studies of disease and environmental effects both on the differentiation of pre-adipocytes into mature adipocytes as well as their impact on the function of mature adipocytes, all derived from the same initial cell population. In addition, adipose spheroids could be maintained in culture for weeks to months allowing for chronic exposure studies to be performed in culture in a high-throughput fashion. Collectively, this report details a valuable *in vitro* tool that can be readily adopted without the need for specialized equipment or materials making an alternative to 2D cultures and animal models accessible to the metabolism and obesity research communities.

## Materials and Methods

### Isolation and culture of pre-adipocytes

Both primary and immortalized cells derived from mouse and human were used in this study. We have previously established and described the immortal normal pre-adipocyte (NPAD) clone B human cell line^25–27^. This cell line was derived from subcutaneous adipose tissue of a non-diabetic donor and immortalized as previously described after informed consent in accordance with a University of Iowa Institutional Review Board approved protocol. It is stable and readily differentiates into mature adipocytes that accumulate lipid droplets and upregulate markers of adipogenesis such as adiponectin, leptin, and fatty acid binding protein (ap2) as well as secrete pro-inflammatory adipokines when stimulated by bacterial toxins.

For primary human pre-adipocytes, subcutaneous fat from non-diabetic donors undergoing surgery for other purposes was collected by the University of Iowa Tissue Procurement Core after informed consent in accordance with a University of Iowa Institutional Review Board approved protocol. Stromal vascular fractions were isolated as previously described^45^. Briefly, fat tissue was digested with type II collagenase (Worthington). Cells were spun down, mature adipocytes (top layer) and supernatant were removed and the pellet was washed 3 times in PBS. The remaining pre-adipocytes were plated in pre-adipocyte growth media (PGM2, Lonza) supplemented with antibiotics. Cells were media changed every day to remove debris and red blood cells. SVF from mouse neonatal visceral or subcutaneous fat was isolated using a procedure similar to that used for human SVF and grown and differentiated as previously described^46^. All mouse experiments presented in this study were conducted in accordance with the animal research guidelines from NIH and were approved by the University of Iowa IACUC.

### Generation of Adipose Spheroids in Hanging Drop Cultures

We used a hanging drop technique to generate 3D spheroids using human pre-adipocytes derived from our immortalized pre-adipocyte cell lines or from primary SVF isolated cells. The size of the spheroids could be tightly controlled by altering the number of cells incorporated into each hanging drop. The spheroids are made by concentrating 5,000 to 20,000 cells in a small volume (5 to 20 µl) of media and placing drops on the inside cover of a petri plate. By using either a multichannel handheld or robotic pipette, hundreds of hanging drops could be made in minutes. The cover was then inverted and placed in a humidified chamber for 2 days to allow cells to coalesce and generate extracellular matrix within the spheroid. After the spheroids formed, they were washed and moved to ultra-low attachment wells with or without differentiation media. Spheroids were either transferred singly to 96-well plates or at indicated multiples in 24-well plates. Care was taken to keep individual spheroids separated to prevent changes in phenotype caused by spheroid fusion.

### Differentiation Media and Spheroid Maintenance

Differentiation media for human cells consisted of PGM2 base media supplemented with a Bullet Kit (Lonza) consisting of indomethacin, dexamethasone, IBMX, and insulin, according the manufacturer’s instructions. Spheroids were maintained in differentiation media or PGM2 base media (non-differentiating media) for indicated times. Media was changed as indicated in the figure legends or, minimally, every 10 days.

### Comparison of 2D and 3D cultures

Equivalent numbers of 2-day old hanging drops were transferred to regular tissue culture plates (2D) or ultra-low adherent plates (3D) in differentiation media. The hanging drops in regular culture plates attached and spread while those in ultra-low attachment plates remained as floating spheroids. Supernatants were collected at indicated time points. The number of cells were quantified using a CyQuant kit according to the manufacturer’s instructions (Invitrogen). Cell numbers were calculated by comparing to a standard curve made with known numbers of cells as counted on a haemocytometer.

### Spheroid Imaging and Histology

Imaging of spheroid size was performed at indicated times using a Nikon Eclipse TE200 microscope with a 10X objective and Nikon ACT-1 software. Volume was calculated by assuming spheroid geometry and using the formula 4/3πr^3^. A minimum of six spheroids were measured for each time point. For histology, spheroids were transferred to an Eppendorf tube and the media was removed. The spheroids were fixed in zinc formalin. The fixed spheroids were embedded in a gelatin/agar solution using a mixture described previously in a tube or OCT cassette^47^. This allowed for paraffin embedding in cassettes by the University of Iowa Comparative Pathology Core. Ten micron sections were cut, deparaffinised and processed for H&E staining. Microscopic images were captured using a Nikon Eclipse E800. Images were then analysed using a CellProfiler^48^ pipeline designed to analyse H&E images in order to quantify the number of nuclei in each spheroid and the number and size of fat droplets in each spheroid (Pipeline in Supplemental Information) For confocal imaging, fixed spheroids were stained with AdipoRed (Lonza) and Hoechst 384 (Sigma). Images were taken using a Leica SP8 STED with Imaris software at the University of Iowa Microscopy Core.

### Adiponectin and Cytokine Release

Supernatants from cell and spheroid cultures were collected at indicated times. ELISAs for adiponectin or IL-8 were performed using ELISA kits (R&D Systems) using dilutions that resulted in values that were detectable within the linear range of the standard curve. A minimum of three biological replicates were assessed for each experimental group.

### Quantitative Reverse-Transcriptase Polymerase Chain Reaction (qRT-PCR)

Spheroids or 2D cultures were homogenized in 1 ml of TRIzol Reagent (Invitrogen). A minimum of 3 spheroids was used to make RNA for each condition. Total RNA from the aqueous phase was further purified using an RNeasy Column (Qiagen). cDNA was synthesized using MMLV Reverse Transcriptase and random Decamers (Invitrogen). Quantitative PCR reactions using SYBR-green master mix (Applied Biosystems) were run on ABI-PRISM sequence Detection System (model 7900HT). After verifying its suitability, beta-actin was used to normalize gene expression. Fold changes compared to untreated controls were calculated using the 2^−DDCt^ method as described^49^. Primers used for the various genes are listed in Table S1.

### High content fluorescent imaging

The hanging drop spheroid method was adapted for high throughput analysis through the use of an ultra-low attachment round bottom spheroid plate (non-adherent 384 well microplate, Cat# 4516, Corning Inc., Corning NY). 500 pre-adipocytes were added to each well and cultured for 11 days in differentiation media. The plate was then mounted in the Operetta High content imaging system (Perkin-Elmer, Waltham, MA) and imaged with 10X LWD objectives automatically for AdipoRed (Lipid droplets) (ex. filter 460–490 nm and em. filter 560–630 nm), Hoechst (nuclei) (ex. filter 360–4000 nm and em. filter 410–480 nm) and Bright light. For confocal (spin disc confocal here on Operetta), the selected wells were imaged with Z-stack of 20 nm interval for 12 stacks. The images were analysed using Harmony software accompanying the instrument. The size and fluorescent intensity of spheroids were calculated for quantification of the differentiation effects.

### Statistics and Presentation of Data

Quantitative data was graphed used GraphPad Prism 7. Statistical analysis was performed with GraphPad Prism using 1-way and 2-way ANOVA depending on the assay. Statistical details are listed in the caption of each figure. For all post-hoc analyses, p < 0.05 was considered statistically significant.

### Data Availability

The datasets generated during and/or analysed during the current study are available from the corresponding authors on reasonable request.

## Electronic supplementary material

Supplementary Information

## References

[1] Nilsson C, Raun K, Yan FF, Larsen MO, Tang-Christensen M (2012). Laboratory animals as surrogate models of human obesity. Acta Pharmacol Sin.

[2] Vickers SP, Jackson HC, Cheetham SC (2011). The utility of animal models to evaluate novel anti-obesity agents. Br J Pharmacol.

[3] Potthoff MJ (2017). FGF21 and metabolic disease in 2016: A new frontier in FGF21 biology. Nature reviews. Endocrinology.

[4] Mlinar B, Marc J (2011). New insights into adipose tissue dysfunction in insulin resistance. Clinical chemistry and laboratory medicine: CCLM/FESCC.

[5] Patel P, Abate N (2013). Role of subcutaneous adipose tissue in the pathogenesis of insulin resistance. Journal of obesity.

[6] Guilherme A, Virbasius JV, Puri V, Czech MP (2008). Adipocyte dysfunctions linking obesity to insulin resistance and type 2 diabetes. Nature reviews. Molecular cell biology.

[7] Rosen ED, Spiegelman BM (2014). What we talk about when we talk about fat. Cell.

[8] Scheid MP, Sweeney G (2014). The role of adiponectin signaling in metabolic syndrome and cancer. Reviews in endocrine & metabolic disorders.

[9] Suzawa M (2003). Cytokines suppress adipogenesis and PPAR-gamma function through the TAK1/TAB1/NIK cascade. Nature cell biology.

[10] Xu H (2003). Chronic inflammation in fat plays a crucial role in the development of obesity-related insulin resistance. The Journal of clinical investigation.

[11] Prentki M, Madiraju SR (2012). Glycerolipid/free fatty acid cycle and islet beta-cell function in health, obesity and diabetes. Molecular and cellular endocrinology.

[12] Wree A, Kahraman A, Gerken G, Canbay A (2011). Obesity affects the liver - the link between adipocytes and hepatocytes. Digestion.

[13] Berry R, Jeffery E, Rodeheffer MS (2014). Weighing in on adipocyte precursors. Cell Metab.

[14] Kusminski CM, Bickel PE, Scherer PE (2016). Targeting adipose tissue in the treatment of obesity-associated diabetes. Nat Rev Drug Discov.

[15] Tchkonia T (2010). Fat tissue, aging, and cellular senescence. Aging cell.

[16] Stanford KI (2015). A novel role for subcutaneous adipose tissue in exercise-induced improvements in glucose homeostasis. Diabetes.

[17] Tran TT, Kahn CR (2010). Transplantation of adipose tissue and stem cells: role in metabolism and disease. Nature reviews. Endocrinology.

[18] Vitseva OI (2008). Inducible Toll-like receptor and NF-kappaB regulatory pathway expression in human adipose tissue. Obesity.

[19] Myre M, Imbeault P (2014). Persistent organic pollutants meet adipose tissue hypoxia: does cross-talk contribute to inflammation during obesity?. Obesity reviews: an official journal of the International Association for the Study of Obesity.

[20] Gourronc, F. A., Robertson, L. W. & Klingelhutz, A. J. A delayed proinflammatory response of human preadipocytes to PCB126 is dependent on the aryl hydrocarbon receptor. *Environ Sci Pollut Res Int*, 10.1007/s11356-017-9676-z (2017). 10.1007/s11356-017-9676-z PMC5764822 28699004

[21] Poulos SP, Dodson MV, Hausman GJ (2010). Cell line models for differentiation: preadipocytes and adipocytes. Experimental biology and medicine.

[22] Toutain, P. L., Ferran, A. & Bousquet-Melou, A. Species differences in pharmacokinetics and pharmacodynamics. *Handb Exp Pharmacol*, 19–48, 10.1007/978-3-642-10324-7_2 (2010). 10.1007/978-3-642-10324-7_2 20204582

[23] Bartosh TJ (2010). Aggregation of human mesenchymal stromal cells (MSCs) into 3D spheroids enhances their antiinflammatory properties. Proc Natl Acad Sci USA.

[24] Frey O, Misun PM, Fluri DA, Hengstler JG, Hierlemann A (2014). Reconfigurable microfluidic hanging drop network for multi-tissue interaction and analysis. Nat Commun.

[25] Vu BG, Gourronc FA, Bernlohr DA, Schlievert PM, Klingelhutz AJ (2013). Staphylococcal superantigens stimulate immortalized human adipocytes to produce chemokines. PloS one.

[26] Littlejohn NK (2016). Suppression of Resting Metabolism by the Angiotensin AT2 Receptor. Cell Rep.

[27] Zhang Y (2017). SWELL1 is a regulator of adipocyte size, insulin signalling and glucose homeostasis. Nature cell biology.

[28] Kwon H, Pessin JE (2013). Adipokines mediate inflammation and insulin resistance. Front Endocrinol (Lausanne).

[29] Gustafson B, Hedjazifar S, Gogg S, Hammarstedt A, Smith U (2015). Insulin resistance and impaired adipogenesis. Trends Endocrinol Metab.

[30] Garcia RA, Roemmich JN, Claycombe KJ (2016). Evaluation of markers of beige adipocytes in white adipose tissue of the mouse. Nutr Metab (Lond).

[31] Linhart HG (2001). C/EBPalpha is required for differentiation of white, but not brown, adipose tissue. Proc Natl Acad Sci USA.

[32] Carmona MC (2002). Mitochondrial biogenesis and thyroid status maturation in brown fat require CCAAT/enhancer-binding protein alpha. J Biol Chem.

[33] Alonso-Magdalena P, Quesada I, Nadal A (2011). Endocrine disruptors in the etiology of type 2 diabetes mellitus. Nature reviews. Endocrinology.

[34] Prajapati B, Jena PK, Rajput P, Purandhar K, Seshadri S (2014). Understanding and modulating the Toll like Receptors (TLRs) and NOD like Receptors (NLRs) cross talk in type 2 diabetes. Curr Diabetes Rev.

[35] Gadupudi G, Gourronc FA, Ludewig G, Robertson LW, Klingelhutz AJ (2015). PCB126 inhibits adipogenesis of human preadipocytes. Toxicol In Vitro.

[36] Schroeder JC (2010). The uremic toxin 3-indoxyl sulfate is a potent endogenous agonist for the human aryl hydrocarbon receptor. Biochemistry.

[37] Tang QQ, Lane MD (2012). Adipogenesis: from stem cell to adipocyte. Annu Rev Biochem.

[38] Abbott RD (2016). The Use of Silk as a Scaffold for Mature, Sustainable Unilocular Adipose 3D Tissue Engineered Systems. Adv Healthc Mater.

[39] Zhang K (2017). Strategy for constructing vascularized adipose units in poly(l-glutamic acid) hydrogel porous scaffold through inducing *in-situ* formation of ASCs spheroids. Acta Biomater.

[40] Louis F (2017). A biomimetic hydrogel functionalized with adipose ECM components as a microenvironment for the 3D culture of human and murine adipocytes. Biotechnol Bioeng.

[41] Verseijden F (2010). Prevascular structures promote vascularization in engineered human adipose tissue constructs upon implantation. Cell Transplant.

[42] Naderi N (2014). Adipogenic differentiation of adipose-derived stem cells in 3-dimensional spheroid cultures (microtissue): implications for the reconstructive surgeon. J Plast Reconstr Aesthet Surg.

[43] Turner PA, Tang Y, Weiss SJ, Janorkar AV (2015). Three-dimensional spheroid cell model of *in vitro* adipocyte inflammation. Tissue Eng Part A.

[44] Wang YH (2014). Characterization and evaluation of the differentiation ability of human adipose-derived stem cells growing in scaffold-free suspension culture. Cytotherapy.

[45] Church CD, Berry R, Rodeheffer MS (2014). Isolation and study of adipocyte precursors. Methods Enzymol.

[46] BonDurant LD (2017). FGF21 Regulates Metabolism Through Adipose-Dependent and -Independent Mechanisms. Cell Metab.

[47] Jones MV, Calabresi PA (2007). Agar-gelatin for embedding tissues prior to paraffin processing. Biotechniques.

[48] Lamprecht MR, Sabatini DM, Carpenter AE (2007). CellProfiler: free, versatile software for automated biological image analysis. Biotechniques.

[49] Livak KJ, Schmittgen TD (2001). Analysis of relative gene expression data using real-time quantitative PCR and the 2(-Delta Delta C(T)) Method. Methods.

