# Supplementary material for: Scaffold-free generation of uniform adipose spheroids for metabolism research and drug discovery
Source: Sci Rep. 2018 Jan 11;8:523. doi: 10.1038/s41598-017-19024-z (PMC5765134; doi:10.1038/s41598-017-19024-z)
Supplement: Supplementary file 1 — Supplementary Information [file 41598_2017_19024_MOESM1_ESM.pdf]

Title: Scaffold-free generation of uniform adipose spheroids for metabolism research and drug discovery.

Authors: Aloysius J. Klingelhutz*[a,b], Francoise A. Gourronc[b], Anna Chaly[b], David A. Wadkins[a,c], Anthony J Burand[a,c], Kathleen R. Markan[a,d], Sharon O. Idiga[a,d], Meng Wu[e,f,g], Matthew J. Potthoff[a,d], James A. Ankrum*[a,c]

***Corresponding Authors:**

james-ankrum@uiowa.edu
Phone: 001-319-335-7512
Fax: 001-319-353-3811
169 Newton Rd, 3336 PBDB
Iowa City, IA 52242

al-klingelhutz@uiowa.edu,
Phone: 001-319-335-7788
Fax: NA
3-612 BSB, 51 Newton Road
Iowa City, IA 52242

Table S1: qRT-PCR primers

	Forward	Reverse	Amplicon size
mADIPOQ	GCA GAG ATG GCA CTC CTG GA	CCC TTC AGC TCC TGT CAT TCC	101 bp
mPPARG	ATT GAG TGC CGA GTC TGT GG	GGC ATT GTG AGA CAT CCC CA	213 bp
mCEBPa	GCC AAG AAG TCG GTG GAC AAG	GTC TCC ACG TTG CGT TGT TTG	110 bp
mFABP4	AAT CAC CGC AGA CGA CAG G	CAT AAC ACA TTC CAC CAC CAG C	142 pb
mCIDEA	GCC GTG TTA AGG AAT CTG CTG	TGC TCT TCT GTA TCG CCC AGT	113 bp
mAct-b	CAT CCT CTT CCT CCC TGG AGA AGA	ACA GGA TTC CAT ACC CAA GAA GGA AGG	125 bp
m18S	AGG GGA GAG CGG GTA AGA GA	GGA CAG GAC TAG GCG GAA CA	241 bp

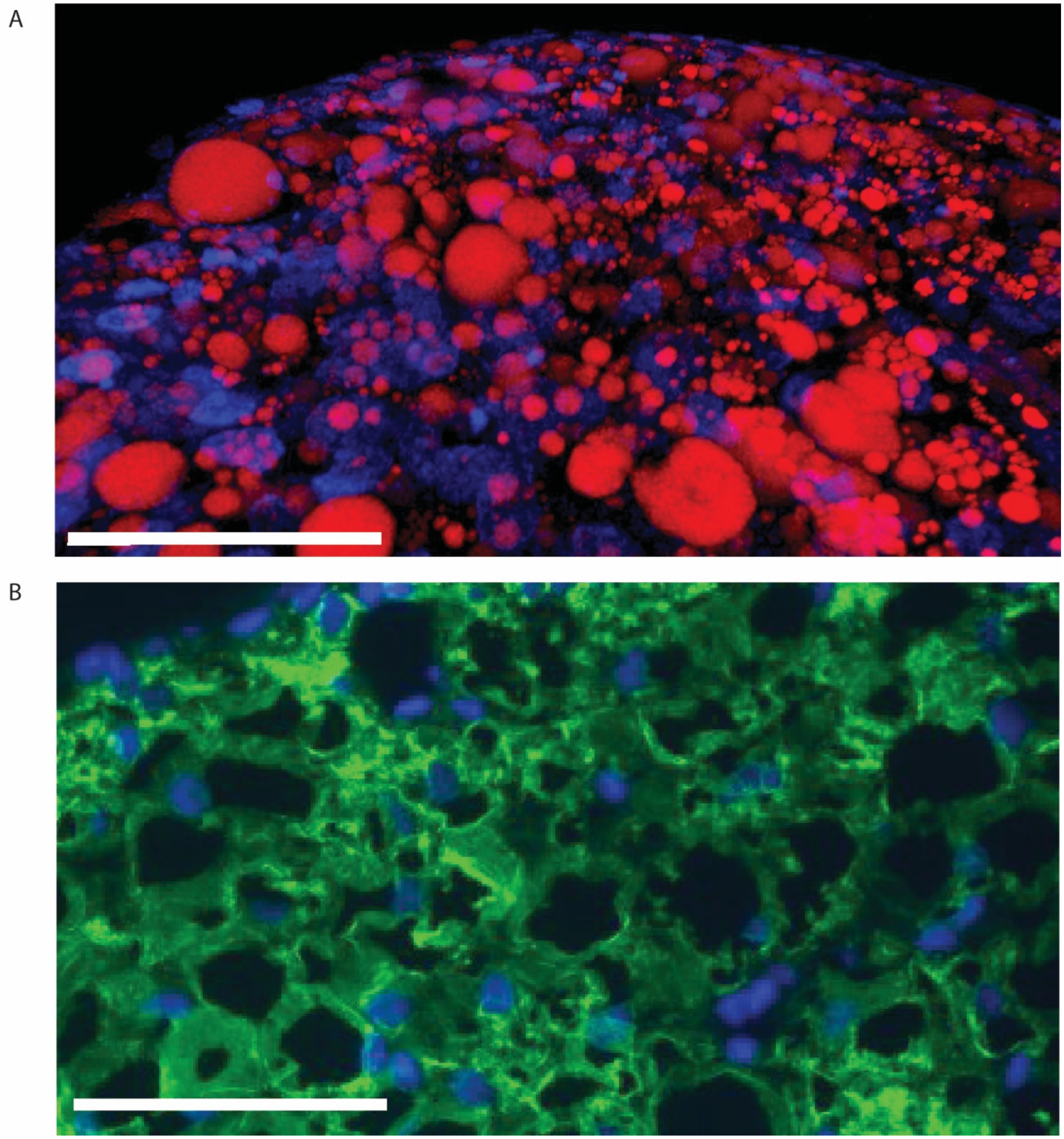

Figure S1: Lipid droplet formation in spheroids. A. Confocal image showing nuclear (Hoechst) and lipid (AdipoRed) staining. A post-day 30 differentiated spheroid was fixed with formalin and stained with Hoechst and AdipoRed. Confocal microscopy was performed using a Leica SP8 STED microscope. The image was processed using Imariz software from Bitplane AG. B. Immunofluorescence image showing perilipin staining. A post-day 30 differentiated spheroid was fixed in formalin and embedded in paraffin. Immunofluorescence was performed on de-parafinized and antigen-retrieved 10 micron sections of the spheroid using a perilipin primary antibody (Abcam ab3526) and a goat anti-rabbit secondary (Alexa 488). Images were taken on an Olympus BX51 microscope. (Scale = 100 μm).
